# Whole-Transcriptome Profiling of Murine GL261 Glioma Reveals Signatures Consistent with Distinct Immune Reprogramming Induced by Oncolytic Virotherapy with VV-GMCSF-Lact and Anti-PD-1 Therapy

**DOI:** 10.3390/ijms27188078

**Published:** 2026-09-11

**Authors:** Anna S. Chesnokova, Alisa B. Ageenko, Natalia S. Vasileva, Arina A. Byvakina, Anna A. Nushtaeva, Anastasia A. Leonteva, Yulya I. Savinovskaya, Galina V. Kochneva, Vladimir A. Richter, Elena V. Kuligina, Dmitriy V. Semenov

**Affiliations:** 1 Institute of Chemical Biology and Fundamental Medicine, Siberian Branch, Russian Academy of Sciences, Lavrentyev Avenue 8, Novosibirsk 630090, Russia; a.ageenko@alumni.nsu.ru (A.B.A.); nataly_vas@bk.ru (N.S.V.); mytrilliangalaxy@gmail.com (A.A.B.); nushtaeva.anna@gmail.com (A.A.N.); anastleont@mail.ru (A.A.L.); yulya_savinovskaya@mail.ru (Y.I.S.); richter@1bio.ru (V.A.R.); kuligina@1bio.ru (E.V.K.); 2 Research Center for Genetics and Life Sciences, Sirius University of Science and Technology, Krasnodar Region, Sirius (Federal Territory of Sirius), Sochi 354340, Russia; 3 State Research Center of Virology and Biotechnology “Vector”, Rospotrebnadzor, Koltsovo 630559, Russia; kochneva@vector.nsc.ru; 4 Oncostar LLC, Inzhenernaya Street 23, Novosibirsk 630090, Russia

**Keywords:** GL261 glioma mouse cell line, C57BL/6 mice, anti-PD-1 therapy, VV-GMCSF-Lact, cancer virotherapy, immune checkpoint inhibitors

## Abstract

Both oncolytic virotherapy and immune checkpoint blockade are being actively explored as immunotherapy treatments for glioblastoma, one of the most lethal malignancies. Nevertheless, the antitumor molecular mechanisms of these therapies remain poorly understood, particularly whether they induce overlapping or distinct transcriptional programs. No comprehensive transcriptome-wide characterization and direct comparison of the responses induced by VV-GMCSF-Lact and anti-PD-1 therapy in glioma have been reported. To address this question, we performed whole-transcriptome profiling of GL261 tumors from immunocompetent C57BL/6 mice treated with VV-GMCSF-Lact, anti-PD-1 antibodies, or their combination. We analyzed therapy-associated changes in gene expression, signaling pathways, and tumor composition. Our findings indicate that anti-PD-1 and combination treatment led to distinctive activation of interferon-gamma response, JAK/STAT signaling, and TNF-alpha/NF-kB pathways. In contrast, VV-GMCSF-Lact preferentially activated T, B, and natural killer T cell-associated programs and reduced the relative abundance of malignant cells. Notably, combination therapy was associated with unique molecular patterns, including induction of monocyte and granulocyte chemotaxis-associated genes, downregulation of PP2A-regulated signaling, and suppression of Rap1-related pathways. This suggests that VV-GMCSF-Lact and anti-PD-1 therapy induce transcriptionally distinct, yet complementary, immune reprogramming and support further evaluation of their combination for glioma immunotherapy.

## 1. Introduction

Gliomas are the most common primary tumors of the brain. Median survival remains poor, at only 12–15 months for patients with glioblastoma—the most aggressive subtype, which also accounts for the largest proportion of glioma cases [1,2].

One of the most promising areas of modern oncology is immunotherapy, which aims to activate the antitumor immune response. Among immunotherapeutic strategies, considerable attention has been given to immune checkpoint inhibition, particularly the blockade of the programmed death-1/programmed death ligand-1 (PD-1/PD-L1) signaling pathway. PD-1 is expressed on T lymphocytes and can limit their cytotoxic activity. Inhibiting the interaction between PD-1 and its ligands can help restore T cell antitumor activity and enhance the antitumor immune response [3].

However, previous studies in murine glioma models have reported that anti-PD-1 monotherapy is insufficient to substantially alter immune cell populations in certain tumor microenvironments (TMEs) and that oncolytic viruses may be required to induce the inflammatory priming necessary for effective checkpoint blockade [4]. It is also important to note that most preclinical studies investigating anti-PD-1 efficacy in glioma models use systemic, typically intraperitoneal, antibody administration, as this route reflects common clinical practice and allows broader antibody interaction with antigen-presenting and immune effector cells beyond the local tumor site [5]. By enhancing tumor immunogenicity, oncolytic virotherapy can also improve responses to immune checkpoint blockade [6,7].

To explore this strategy as a combination immunotherapy for glioma, we employ a previously developed recombinant vaccinia virus strain VV-GMCSF-Lact. This strain contains deletions of the viral thymidine kinase and vaccinia growth factor genes, which are replaced with the human GM-CSF and lactaptin genes. Lactaptin, a fragment of human milk kappa-casein, exhibits potent pro-apoptotic and antitumor activity, while GM-CSF enhances antitumor immune responses. In our previous studies, VV-GMCSF-Lact demonstrated strong cytotoxic and antitumor effects in both immortalized glioblastoma cell lines and patient-derived glioblastoma cultures [8]. However, transcriptomic analyses revealed activation of the PD-1/PD-L1 signaling pathway following VV-GMCSF-Lact treatment [6], suggesting that immune checkpoint activation may limit the therapeutic efficacy of the virus. These findings provide a rationale for combining oncolytic viruses with immune checkpoint inhibitors to enhance antitumor immune responses.

In this study, we performed whole-transcriptome analysis of tumors collected from C57BL/6 mice bearing subcutaneous GL261 gliomas following treatment with the oncolytic virus VV-GMCSF-Lact, anti-PD-1 monoclonal antibodies, or their combination. We aimed to identify transcriptomic changes underlying the antitumor effects of these therapies and to characterize the associated remodeling of the TME.

VV-GMCSF-Lact and anti-PD-1 monotherapies induced distinct transcriptomic programs. Their combination retained transcriptional features of both while also inducing unique gene expression changes not observed with either treatment alone. Anti-PD-1 antibodies were the primary drivers of global transcriptional remodeling, promoting activation of IFNG- and NF-kB-related signaling pathways. In contrast, VV-GMCSF-Lact uniquely enhanced T and B cell-associated transcriptional programs and reduced the estimated malignant cell fraction. Notably, the combination treatment induced additional transcriptional changes not observed with either monotherapy, including the upregulation of genes involved in monocyte chemotaxis. Overall, these findings support further investigation of PD-1 blockade combined with VV-GMCSF-Lact as a therapeutic strategy for glioma.

## 2. Results

**Figure 1:**
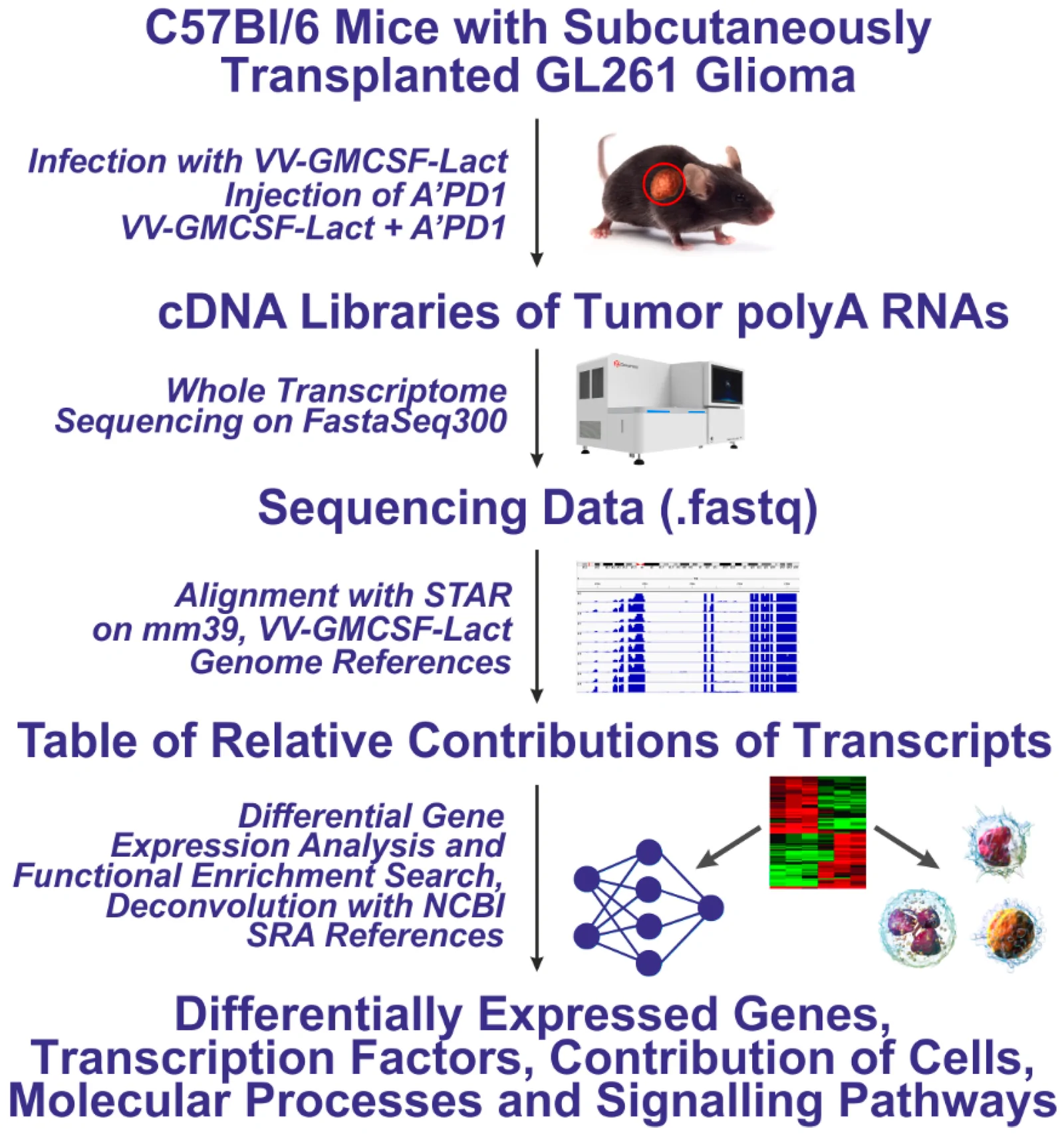
Experimental scheme for evaluating the effects of anti-PD-1 antibodies (A’PD1), the oncolytic virus VV-GMCSF-Lact, and their combination (VV-GMCSF-Lact + A’PD1) on GL261 glioma tumors subcutaneously transplanted into immunocompetent C57BL/6 mice.

To investigate the molecular processes underlying the antitumor effects of VV-GMCSF-Lact and anti-PD-1 therapy, we transplanted murine GL261 glioma cells into immunocompetent C57BL/6 mice. The established tumors were treated by intratumoral injection of VV-GMCSF-Lact, intraperitoneal administration of anti-PD-1, or their combination. After completing therapy, the tumors were removed, and total RNA was isolated from tumor samples. We then prepared whole-transcriptome cDNA libraries from the polyA fraction of total tumor RNA. Sequencing data (GeneMind FastaSeq300) were aligned to the mouse reference genome assembly GRCm39/mm39 and the VV-GMCSF-Lact genome using STAR [9]. Differential gene expression analysis was performed using DESeq2 [10]. Functional enrichment analysis of differentially expressed gene sets was conducted using Enrichr [11]. To evaluate therapy-associated remodeling of the tumor cellular composition, deconvolution analysis was performed using DeconRNASeq [12] with a curated reference panel of 54 mouse cell-type transcriptomes retrieved from the NCBI SRA database, covering immune cell populations, stromal components, and GL261 glioma reference transcriptomes (Figure 1, detailed in Section 4.3).

### 2.1. Global Transcriptome Overview of GL261 Tumors

RNA-seq analysis of 8 cDNA libraries generated approximately 13–25 million reads per sample, with over 95% of reads successfully aligned to the mouse genome (mm39) using STAR (Table 1).

**Figure 2:**
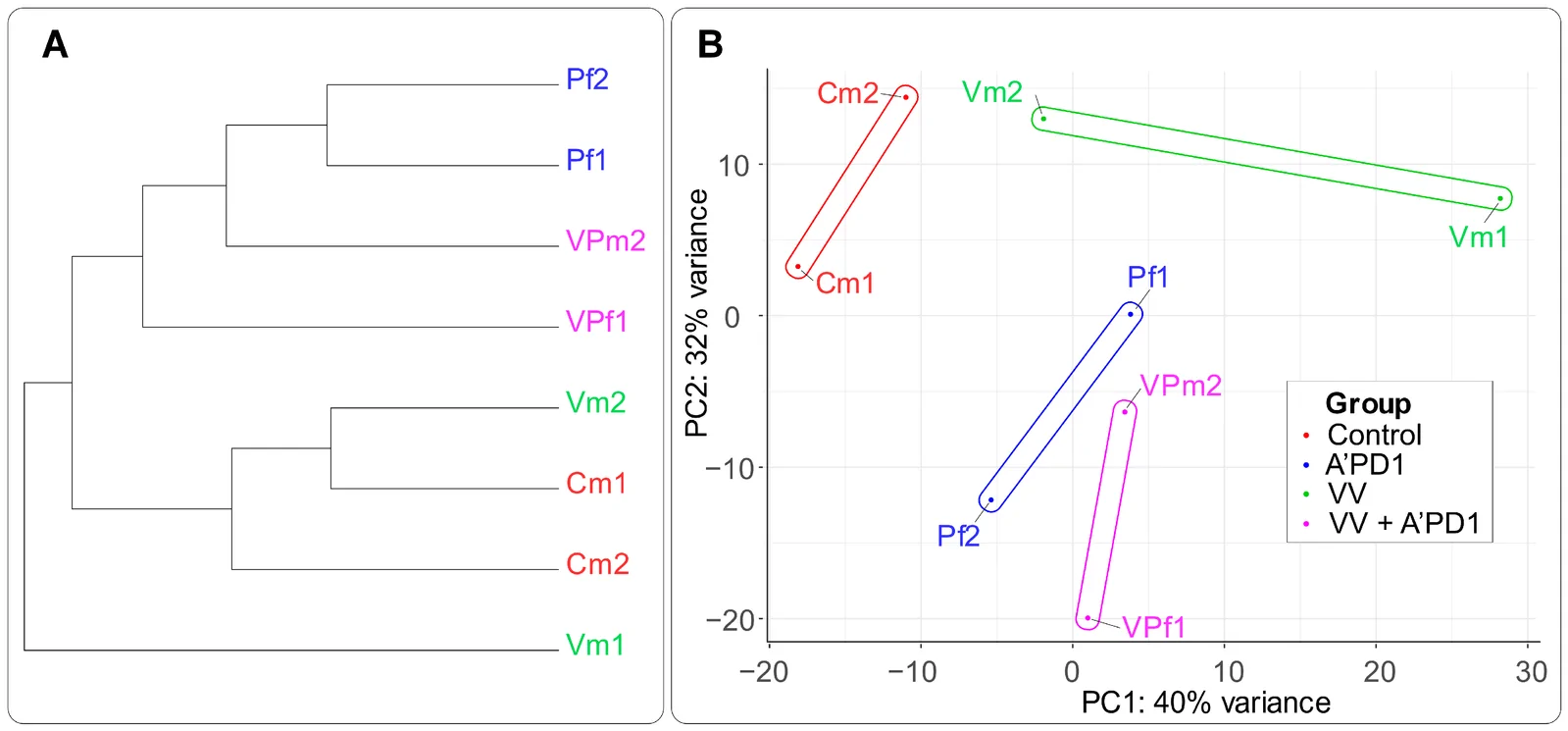
(**A**) Hierarchical clustering of transcriptomes from GL261 glioma tumors subcutaneously transplanted into C57BL/6 mice. (**B**) Principal component analysis (PCA) of tumor transcriptomes with linear dimensionality reduction in PC1:PC2 space. The same color scheme was used in panels (**A**,**B**) to indicate the different post-treatment groups: Pf(1/2)—anti-PD-1 antibodies; Vm(1/2)—VV-GMCSF-Lact; VP(f1/m2)—combination of VV-GMCSF-Lact and anti-PD-1 antibodies; Cm(1/2)—control tumors. “f” denotes female mice, and “m” denotes male mice.

GL261 glioma transcriptomes clustered into three distinct branches: (1) after therapy with anti-PD-1 and the anti-PD-1 + VV-GMCSF-Lact combination; (2) a mixed group of tumor samples from control animals and tumors treated with the oncolytic virus; and (3) the Vm1 sample (Figure 2A).

Principal component analysis (PCA) showed that the transcriptomes of control and treated tumors formed distinct, non-overlapping clusters in the PC1:PC2 space. Notably, the PC1:PC2 shift vectors were oriented in opposite directions for tumors treated with the oncolytic virus compared to those treated with anti-PD-1 alone or in combination with the virus (Figure 2B).

Overall, whole-transcriptome RNA-seq analysis demonstrated high sequencing and alignment quality across all tumor samples, providing a reliable basis for comparative gene expression analysis. Hierarchical clustering and PCA revealed that anti-PD-1 therapy, either alone or in combination with VV-GMCSF-Lact, induced coordinated transcriptomic remodeling in GL261 glioma tumors, as reflected by clear separation of these groups from control tumors. In contrast, tumors treated with VV-GMCSF-Lact alone clustered closer to control samples, indicating a less extensive transcriptional response. Moreover, the VV-GMCSF-Lact-treated samples had greater heterogeneity: Vm2 remained relatively close to the control group, whereas Vm1 showed a much larger displacement along the PC1 axis. Together, these findings indicate that immune checkpoint blockade is the primary driver of transcriptomic reprogramming in this model, whereas oncolytic virotherapy alone elicits a distinct and more heterogeneous molecular response.

### 2.2. Deconvolution Analysis

**Figure 3:**
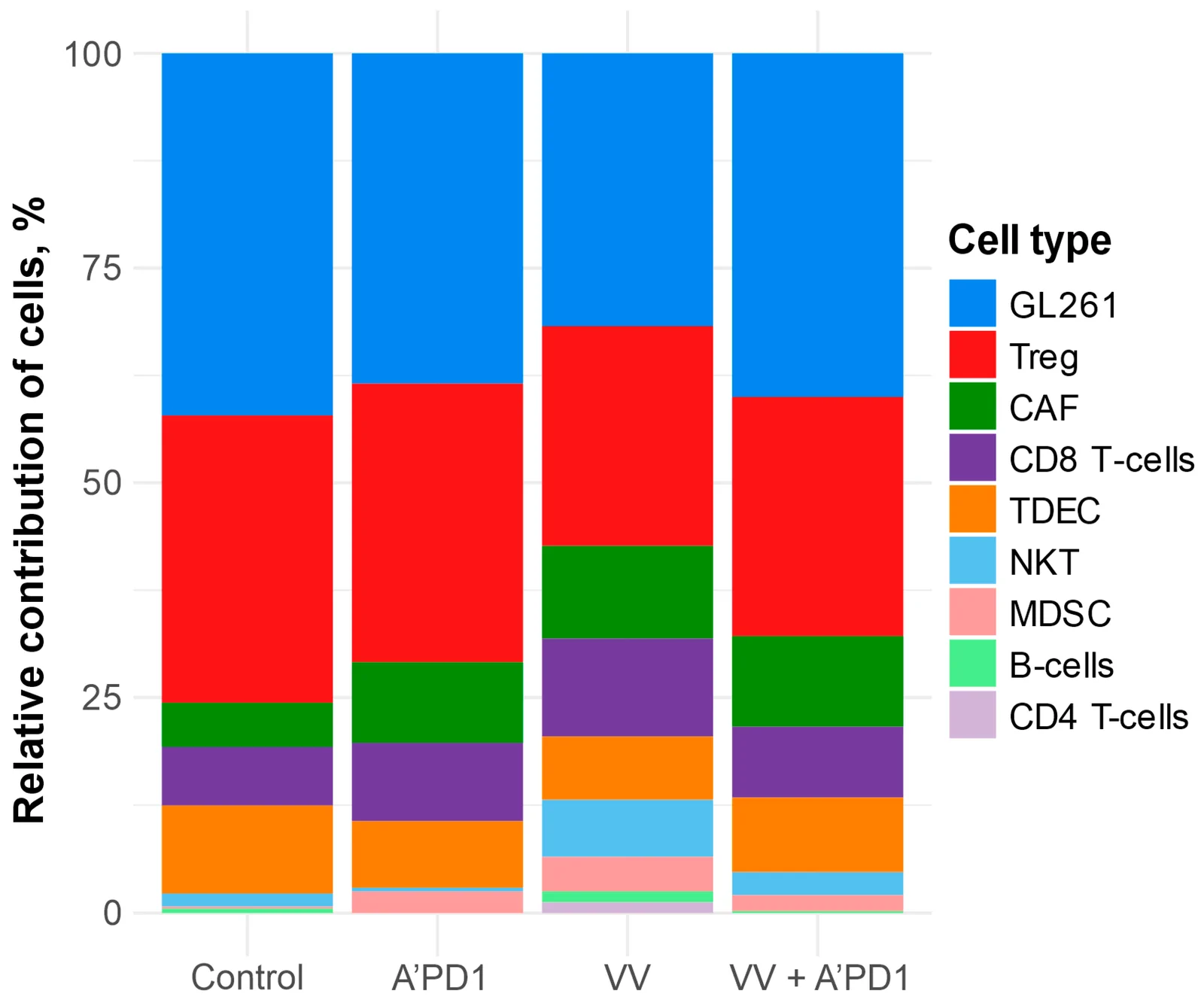
Changes in the cellular composition of subcutaneously transplanted glioma tumors in mice following treatment with the oncolytic virus VV-GMCSF-Lact (VV), anti-PD-1 antibodies (A’PD1), or their combination (VV + A’PD1). Data were obtained using DeconRNASeq V1.50.0 and reference transcriptome arrays of mouse cells from the NCBI SRA archive (Table S2). The legend and list of SRA identifiers for the final set of reference transcriptomes are provided in Table S1.

To explore potential therapy-associated changes in the transcriptional representation of tumor and host cell types, we performed transcriptome deconvolution analysis [12]. The estimated relative contribution of the GL261 transcriptional program decreased from 42% in control tumors to 31% in VV-GMCSF-Lact-treated tumors (Figure 3, Table S1, “GL261”). Both viral infection and combination therapy were associated with an increased estimated representation of the cancer-associated fibroblast transcriptional program, from 5% to 11% (Table S1, “CAF”).

Overall, deconvolution analysis indicated that therapy-induced remodeling of the TME in GL261 gliomas was primarily associated with the oncolytic virus VV-GMCSF-Lact. Viral therapy reduced the relative contribution of GL261 tumor cells and was accompanied by increased representation of stromal and immune-associated cell populations, including CAFs, CD8 T cells, NKT cells, MDSCs, B cells, and CD4+ T cells. At the same time, the relative abundance of Treg cells decreased in virus-treated tumors. Combination therapy induced similar, although less pronounced, changes, including a reduction in Treg cells and an increase in CAFs and NKT cells, whereas anti-PD-1 monotherapy led to comparatively minor alterations in tumor cellular composition. These trends suggest that VV-GMCSF-Lact treatment alters the estimated composition of the TME, potentially reflecting shifts in immune and stromal transcriptional programs within transplanted gliomas. Importantly, these changes were observed despite the weaker transcriptomic shift from control induced by the oncolytic virus compared with other therapy modes, as shown by hierarchical clustering and PCA (Figure 2B).

### 2.3. Differential Gene Expression Analysis

Differential gene expression analysis was performed to identify signaling pathways, transcriptional regulators, and cellular processes associated with VV-GMCSF-Lact and anti-PD-1 treatment in glioma.

**Figure 4:**
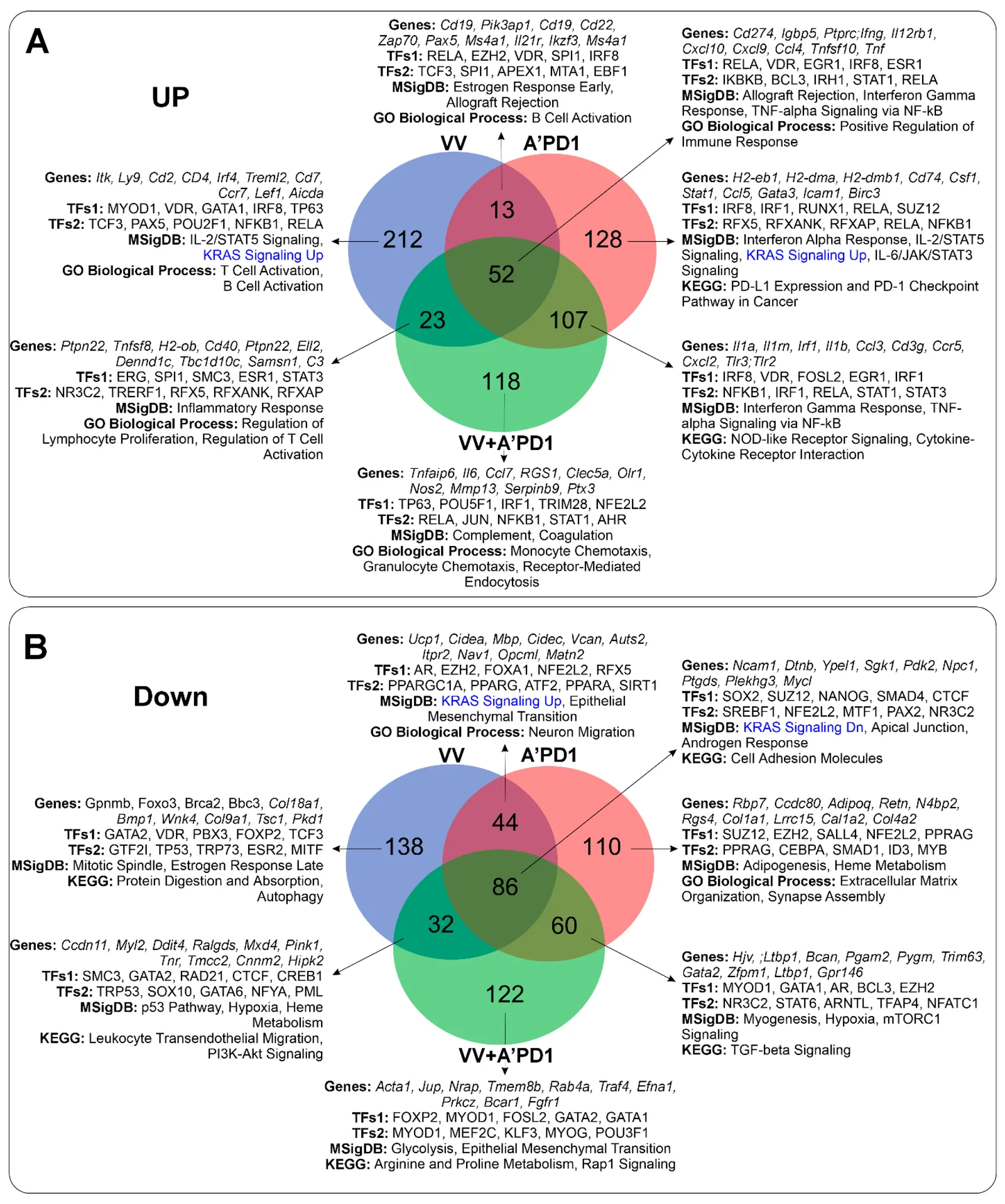
Venn diagrams showing the overlap among the top 300 upregulated (**A**) and downregulated (**B**) transcripts, identified by comparing control glioma tumors with tumors treated with VV-GMCSF-Lact (VV), anti-PD-1 antibodies (A’PD1), or their combination (VV + A’PD1). Selected lists of characteristic differentially expressed transcripts (Genes) are shown together with representative results from Enrichr analysis using the following libraries: TF1—“ENCODE and ChEA Consensus TFs from ChIP-X”; TF2—“TRRUST Transcription Factors 2019”; MSigDB—“MSigDB Hallmark 2020”; “GO Biological Process 2025”; KEGG—“KEGG 2021 Human”, “KEGG 2019 Mouse”.

We compiled lists of the top 300 differentially expressed transcripts (adjusted *p*-value < 0.05; |log2FoldChange| > 0.5). As shown in Figure 4, comparison of the upregulated transcript sets identified 52 transcripts that were consistently increased across all treatments: VV-GMCSF-Lact, anti-PD-1 antibodies, and their combination. Enrichr analysis of this shared gene set using the “ENCODE and ChEA Consensus TFs from ChIP-X” library implicated transcription factors (TFs) including RELA, VDR, EGR1, IRF8, and ESR1. The gene set was also predicted to be regulated by IKBKB, BCL3, IRF1, STAT1, and RELA, as determined using the Enrichr “TRRUST Transcription Factors 2019” library (Figure 4A). The key feature of these genes is their involvement in allograft rejection, interferon-gamma response, and TNF-alpha signaling via NF-kB pathways (Figure 4A, “MSigDB”).

Importantly, among the top 300 differentially expressed genes, the greatest overlap was observed between the anti-PD-1 and combination therapy groups (107 shared transcripts, compared to 13 and 23 transcripts in the other pairwise intersections; Figure 4A). This is consistent with the global transcriptome analysis, in which tumors treated with combination therapy showed a transcriptional profile more closely resembling that of anti-PD-1 monotherapy than that of VV-GMCSF-Lact treatment (Figure 2).

Analyzing transcripts uniquely upregulated in each treatment group, we also identified distinct biological signatures. VV-GMCSF-Lact treatment was associated with pathways related to T and B cell activation in response to viral infection. Anti-PD-1 monotherapy preferentially enriched interleukin signaling pathways and, notably, PD-1 expression and signaling in cancer. In contrast, combination therapy uniquely enriched pathways involved in the modulation of monocyte and granulocyte chemotaxis (Figure 4A, “GO Biological Process 2025” and “MSigDB Hallmark 2020”).

Next, we compared the 300 most downregulated transcripts and identified 86 common mRNAs that were consistently decreased across all treatment groups. This shared transcript set was enriched for mRNA-encoding proteins involved in KRAS signaling, apical junction organization, and androgen response (Figure 4B). Importantly, treatment with anti-PD-1 antibodies, the oncolytic virus VV-GMCSF-Lact, or their combination independently suppressed the epithelial–mesenchymal transition (EMT) signature (Figure 4B, “Epithelial–Mesenchymal Transition”).

Among uniquely downregulated transcripts in each treatment group, the following pathways could be singled out: spindle formation and late estrogen receptor response genes for VV-GMCSF-Lact treatment; extracellular matrix organization and adipogenesis for anti-PD-1 monotherapy; glycolysis, arginine, and proline metabolism; and the Rap1 signaling pathway for combination therapy (Figure 4B).

Our data showed that both the oncolytic virus and anti-PD-1 independently increase the mRNA level of genes upregulated by KRAS activation (Figure 4A, “KRAS Signaling Up”). Moreover, transcripts comprising genes downregulated by KRAS activation were consistently downregulated across all treatment groups (Figure 4B, “KRAS Signaling Dn”).

In summary, transcriptomic profiling of GL261 gliomas revealed that VV-GMCSF-Lact and anti-PD-1 antibodies induce distinct gene expression programs when administered either as monotherapies or in combination. All three treatment regimens shared a core set of 52 commonly upregulated transcripts involved in interferon-gamma and TNF-alpha/NF-kB signaling and regulated by TFs EGR1, RELA, IRF1, and STAT1. Specifically, virus therapy preferentially induced pathways associated with T and B cell activation, whereas anti-PD-1 therapy selectively enriched interleukin and PD-1 signaling, and combination therapy uniquely modulated monocyte/granulocyte chemotaxis. In addition, despite these differences, all three treatments consistently suppressed EMT and modulated KRAS signaling transcripts.

### 2.4. Functional Analysis of Molecular Pathways

It has previously been shown that the oncolytic virus VV-GMCSF-Lact activates pathways associated with immune checkpoint inhibitors and the transcription factor NF-kB [6,7,13]. To illustrate how the different therapies modulate the expression of genes associated with these pathways, we generated a signaling schematic based on published literature [14,15,16,17,18].

**Figure 5:**
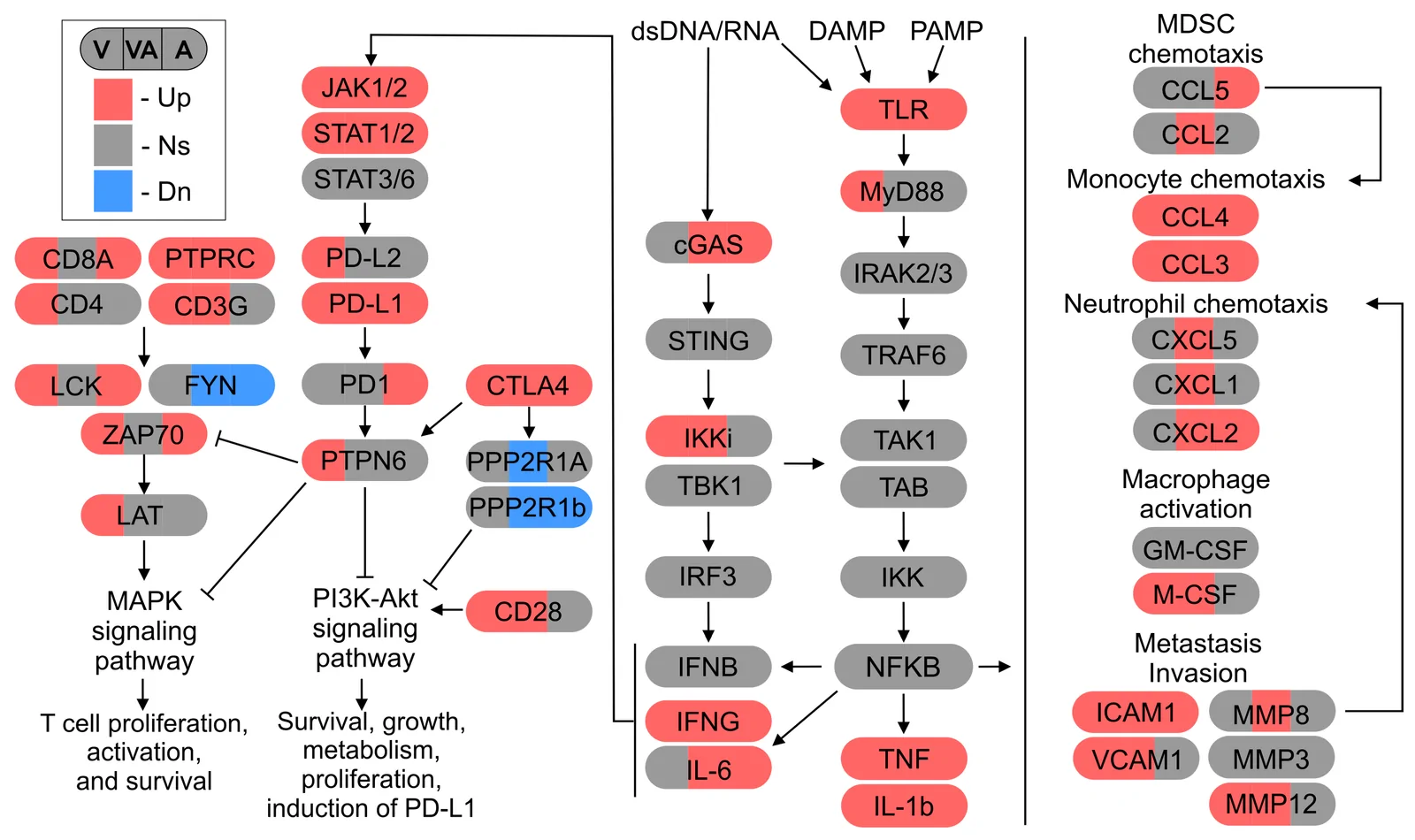
Literature-derived molecular processes and signaling pathways, annotated with predicted activation or suppression based on transcript-level changes in GL261 tumors following treatment with VV-GMCSF-Lact, anti-PD-1 antibodies, or combination therapy. Differentially expressed genes are designated as follows: V—VV-GMCSF-Lact; A—A’PD1 antibodies; VA—combination therapy. Up—increased expression (red); Dn—decreased expression (blue) (adjusted *p*-value < 0.05, |log2FoldChange| > 0); Ns—no significant differential expression (grey).

Across all treatment groups, expression of the interferon gene *Ifng*, the pro-inflammatory cytokine genes (*Il1b*, *Tnf*), and the signaling molecules *Jak1*, *Jak2*, *Stat1*, and *Stat2* was consistently increased (Figure 5), which is in agreement with the transcriptome-wide analyses presented in Section 2.3 (Figure 4A).

Treatment-specific differences were also observed. Anti-PD-1 therapy increased *Pd1* mRNA levels, consistent with the enrichment analysis in Section 2.3 (Figure 4A), whereas VV-GMCSF-Lact treatment selectively upregulated *Pd-l2* mRNA. Elevated *Pd-l1* expression was detected in all treatment groups (Figure 5). Increased *Ptpn6* mRNA expression was observed only after VV-GMCSF-Lact treatment. *Ctla4* expression was higher after each therapeutic intervention, and reduced *Ppp2r1a/b* expression was detected only in the combination therapy group.

Among genes associated with T cell activation signaling, VV-GMCSF-Lact predominantly increased the expression of *Cd8a*, *Cd4*, *Lck*, *Zap70*, and *Lat*. In contrast, increased expression of *Cd28* and *Cd3g* was observed after both VV-GMCSF-Lact treatment and combination therapy (Figure 5).

Analysis of chemokine-related genes demonstrated therapy-dependent modulation of pathways involved in immune cell recruitment. Upregulation of *Ccl2*, *Cxcl1*, *Cxcl5*, and *Mmp8* was detected exclusively in tumors treated with combination therapy. Additionally, combination therapy retained the upregulation of *Vcam*, *M-csf*, *Cxcl2*, and *Mmp12* observed following oncolytic virus or anti-PD-1 monotherapy. Finally, elevated expression of *Ccl3* and *Ccl4* was observed in all three treatment groups (Figure 4A and Figure 5).

## 3. Discussion

Glioblastoma remains one of the most aggressive and treatment-resistant malignancies despite advances in surgery, radiotherapy, and chemotherapy [1]. A major obstacle to successful therapy is the highly immunosuppressive TME, which limits the efficacy of immunotherapeutic approaches such as immune checkpoint inhibitors [4]. Although oncolytic viruses have been shown to stimulate antitumor immunity and remodel the TME, the molecular mechanisms underlying their interaction with immune checkpoint blockade remain unclear [6]. Therefore, it is essential to characterize transcriptomic changes induced by virotherapy, anti-PD-1 treatment, and their combination in order to understand the biological basis of therapeutic responses and to identify mechanisms that could inform the rational design of combination strategies for glioma.

In this study, we performed whole-transcriptome analysis of subcutaneously transplanted GL261 glioma tumors treated with the oncolytic vaccinia virus VV-GMCSF-Lact, anti-PD-1 antibodies, or their combination. We aimed to characterize the transcriptome changes mediating the antitumor activity of each therapeutic strategy and to map the associated changes in the TME.

Hierarchical clustering and PCA of the transcriptome data revealed that anti-PD-1 therapy induced a coherent transcriptomic reprogramming in GL261 tumors, clearly separating these groups from the control. In combination with VV-GMCSF-Lact, the transcriptomic profile was dominated by the anti-PD-1 signature. In contrast, VV-GMCSF-Lact monotherapy elicited a more heterogeneous response, with treated tumors clustering closer to the control group (Figure 2). The opposite directions of the PCA shift vectors for the virus-treated and anti-PD-1-containing treatment groups may reflect distinct transcriptomic responses.

The significant overlap between the anti-PD-1 and combination therapy transcriptomes supports this interpretation. Of the top 300 upregulated transcripts, 107 were shared between these two groups, whereas only 13–23 transcripts overlapped in the other pairwise intersections (Figure 4). This indicates that in the subcutaneous GL261 model, immune checkpoint blockade via PD-1 is the dominant driver of transcriptomic remodeling, whereas the oncolytic virus induces a distinct set of biological processes. Notably, acting directly within the TME, intratumorally delivered VV-GMCSF-Lact might be expected to produce a more pronounced local transcriptional effect than systemically administered anti-PD-1. Instead, our data suggest that PD-1 blockade-driven remodeling is not simply a function of local drug concentration at the tumor site. This pattern is consistent with the broader preclinical literature, where intraperitoneal anti-PD-1 administration reliably induces robust antitumor transcriptional and immune effects in glioma models [5].

Nevertheless, combination therapy also produced unique transcriptional changes that neither monotherapy induced. Most notably, it upregulated genes involved in monocyte chemotaxis (*Ccl2*, *Cxcl1*, *Cxcl5*, and *Mmp8*), which suggests that the two modalities may interact and generate novel biological signals when used together.

Among transcripts that increased in all three treatment groups, the most enriched hallmark gene sets included interferon-gamma response, TNF-alpha signaling via NF-kB, and allograft rejection. Transcription factor enrichment analysis identified RELA, IRF1, STAT1, and other NF-kB pathway components as the principal regulators of this shared transcriptional program (Figure 5).

All treatments also increased the expression of *Ifng*, *Il1b*, and *Tnfa*, indicating a common proinflammatory shift within the TME regardless of therapeutic modality (Figure 4A). This response may reflect a shared innate immune response to tumor cell lysis and immune activation. These findings are consistent with previous studies showing that anti-PD-1 therapy converges on NF-kB and interferon signaling as central regulatory nodes [4,6]. We have also previously shown that infection of human cells with VV-GMCSF-Lact increases the expression of genes regulated by NF-kB, potentially through innate antiviral responses mediated by the cGAS–STING and TLR-dependent signaling pathways [6].

Notably, PD-L1 (Cd274) mRNA expression was elevated following all three treatments as well, whereas only VV-GMCSF-Lact upregulated PD-L2 (Figure 5). The upregulation of PD-L1 in response to IFNG is a well-characterized feedback mechanism of adaptive immune resistance in glioma. IFNG released by tumor-infiltrating CD8 T cells activates the JAK1/JAK2–STAT1–IRF1 axis, which in turn drives PD-L1 transcription in both tumor and stromal cells, suppressing antitumor immunity. Studies in glioblastoma have confirmed this mechanism: IFNG increases phosphorylated STAT1 levels, upregulates IRF1 and PD-L1, and may promote tumor cell migration [14,19]. Importantly, persistent activation of STAT1/IRF1 signaling can also promote acquired resistance to PD-(L)1 blockade through epigenetic stabilization of T cell exhaustion [20]. This allows us to suggest that IFNG upregulation observed in our study may play a dual role: it may transiently enhance antitumor immunity while simultaneously creating conditions that favor adaptive resistance. Taken together, these findings identify the JAK/STAT1/IRF1/PD-L1 axis as a central pathway that links virus-induced and checkpoint-mediated inflammation in the glioma TME [15].

The concurrent increase in IFNG and PD-L1 across all treatment groups (Figure 5) supports this feedback loop model and provides a mechanistic rationale for combining anti-PD-1 therapy with oncolytic virotherapy. Viral infection promotes T cell infiltration and IFNG release, which drives PD-L1 upregulation, while checkpoint blockade helps prevent subsequent T cell exhaustion [21]. Increased PD-1 mRNA expression after anti-PD-1 treatment may be associated with compensatory receptor upregulation in response to antibody-mediated blockade, a phenomenon also reported in other tumor models [22].

All three treatment strategies suppressed the expression of genes associated with epithelial–mesenchymal transition (EMT; Figure 4). As EMT facilitates tumor invasiveness, therapy resistance, and immunosuppression in glioblastoma, this transcriptional response may reflect a reduced capacity for tumor cell migration and immune escape [23]. In addition, all treatments affected KRAS-associated transcriptional programs, although the direction of these changes differed between treatment-specific and shared gene signatures, indicating context-dependent remodeling of pathways linked to KRAS signaling. Given the central role of KRAS-regulated networks in controlling glioma cell proliferation, survival, and immune interactions [24], these findings suggest that immune-based therapies broadly reshape KRAS-associated transcriptional responses rather than uniformly activating or suppressing this signaling axis. Together, the suppression of EMT and modulation of KRAS-associated transcriptional programs may represent a common feature of GL261 glioma tumors’ response to immune-mediated pressure, irrespective of the therapeutic modality.

VV-GMCSF-Lact monotherapy uniquely increased the expression of genes involved in T and B cell activation and NKT cell signaling and upregulated T cell activation markers, including *Cd8a*, *Cd4*, *Lck*, *Zap70*, *Lat*, and *Cd28* (Figure 5). These findings suggest that intratumoral virus delivery directly stimulates adaptive immune responses within the tumor. This is consistent with the well-established ability of vaccinia viruses to act as potent immunological adjuvants and to convert immunologically cold tumors into inflamed, lymphocyte-infiltrated lesions [6,8].

Deconvolution analysis was broadly consistent with these transcriptomic observations, suggesting higher estimated proportions of CD8^+^ T cell, NKT cell, and B cell transcriptional programs in virus-treated tumors, alongside a lower estimated representation of the GL261 transcriptional program (Figure 3, Table S1). The decrease in the estimated fraction of glioma cells was accompanied by reduced expression of genes involved in mitotic spindle assembly and EMT-related programs, suggesting that the treatment attenuated both proliferative and invasive tumor phenotypes. This decrease most likely reflects direct viral cytolysis, as previously demonstrated in vitro and in vivo for VV-GMCSF-Lact in GL261 and other glioma models [6,8].

Tumors from virus-treated mice and those receiving combination therapy also showed an increased proportion of cancer-associated fibroblasts (CAFs) (Figure 3). This change may reflect reactive stromal remodeling in response to virus-induced tumor necrosis. Because CAFs can either promote or restrain antitumor immunity [25], their role in this process requires further investigation.

Only combination therapy was found to increase the expression of the monocyte chemotaxis-associated genes *Ccl2*, *Cxcl1*, *Cxcl5*, and *Mmp8*, suggesting that the two treatments engage complementary immune processes. CCL2, CCL3, and CCL4 are key chemokines that recruit monocytes and macrophages to the tumor and influence the polarization of the myeloid compartment within the TME. In glioma, the balance between immunostimulatory and immunosuppressive myeloid cells is a critical determinant of therapeutic outcome [16]. The combination of virus-induced T cell activation and selective induction of monocyte-recruiting chemokines therefore suggests that VV-GMCSF-Lact and anti-PD-1 together may contribute to a broader immune activation program that coordinates both the adaptive and innate immune compartments. The biological significance of this coordinated immune response, and whether it translates into improved tumor control, remains to be established.

Combination therapy also selectively reduced *Ppp2r1a* expression, whereas both A’PD1 monotherapy and combination therapy reduced *Ppp2r1b* expression (Figure 5). *Ppp2r1a* and *Ppp2r1b* encode regulatory subunits of the tumor suppressor protein phosphatase 2A (PP2A). Their downregulation can serve a dual function: on the one hand, PP2A suppresses uncontrolled growth and limits malignant progression [26], and on the other hand, it acts as an intracellular ‘brake’ on T lymphocytes by reducing their survival, proliferation, and cytotoxic activity against cancer [27].

VV-GMCSF-Lact treatment increased *Ptpn6* expression, which may indicate activation of inhibitory phosphatase-mediated signaling linked to PD-1-dependent regulation of the MAPK and PI3K-Akt pathways [17]. In parallel, elevated *Ctla4* expression was detected following all three therapeutic interventions. CTLA4-associated signaling is known to suppress T cell activation by recruiting phosphatases, including the PP2A complex components [28]. However, the reduced expression of *Ppp2r1a/b* in anti-PD-1 and combination therapy may partially offset this inhibitory signaling in activated immune cells.

Finally, combination therapy was also associated with downregulation of the Rap1 signaling pathway. Given the shared suppression of EMT-associated genes and the established role of Rap1 signaling in cell migration, adhesion, and glioma invasiveness [18], combined treatment may more effectively attenuate pro-invasive tumor programs than either monotherapy alone.

The distinct transcriptional programs induced by combination therapy highlight the complexity of interactions between oncolytic virotherapy and immune checkpoint blockade.

Several limitations of this study should be noted. Due to the small sample size inherent to this exploratory transcriptome analysis, our results do not allow us to correlate tumor transcriptome changes with animal sex. Future studies using orthotopic glioma models, balanced sex representation, a higher number of biological replicates, and orthogonal validation of key findings will be needed to confirm these transcriptional patterns and their relevance to glioma immunotherapy.

## 4. Materials and Methods

### 4.1. Glioma Cell Cultures

Murine GL261 glioma cells (DSMZ ACC-802), kindly provided by Alexey Stepanenko (Department of Neurobiology, Serbsky National Medical Research Center for Psychiatry and Narcology, Moscow, Russia), were cultured in Dulbecco’s Modified Eagle Medium (DMEM; Thermo Fisher Scientific, Waltham, MA, USA) supplemented with 10% FBS (Thermo Fisher Scientific, Waltham, MA, USA), 2 mM L-glutamine (Invitrogen, Waltham, MA, USA), and antimycotic/antibiotic solution (100 U/mL penicillin, 100 mg/mL streptomycin sulfate, 0.25 μg/mL amphotericin; Sigma-Aldrich, Darmstadt, Germany) in 25 cm^2^ culture flasks (TPP, Trasadingen, Switzerland). Cells were maintained at 37 °C in a humidified 5% CO_2_ atmosphere to promote cell adhesion.

### 4.2. Experimental Animals

All animal procedures were performed at the Institute of Chemical Biology and Fundamental Medicine, Siberian Branch of the Russian Academy of Sciences (SB RAS; Novosibirsk, Russia). All experiments with laboratory animals were carried out in accordance with the recommendations and requirements of the World Society for the Protection of Animals (WSPA) and the European Convention for the Protection of Experimental Animals (Strasbourg, 1986) [29], as well as the ARRIVE guidelines. The study was approved by the Bioethics Committee of the Institute of Cytology and Genetics SB RAS (Protocol No. 197, 21 November 2024).

The study included 5 male and 3 female C57BL/6 mice (n = 8; body weight 18–20 g) obtained from the SPF Vivarium of the Institute of Cytology and Genetics SB RAS (Novosibirsk, Russia). The sample size was determined primarily by the 3Rs principle of Reduction, given the exploratory nature of this first transcriptomic comparison across three treatment arms and a control group, rather than by an a priori power calculation. This is acknowledged as a limitation of the study (see Section 3). Mice were housed in groups in standard cages under a 12 h light/dark cycle at 20–24 °C and 45–55% relative humidity. Water and standard chow were provided ad libitum.

### 4.3. GL261 Glioma Transplantation and Treatment

GL261 glioma cells were transplanted subcutaneously into C57BL/6 mice. Tumor size was monitored using caliper measurements. Once tumors reached a volume of 120–150 mm^3^, mice were randomized into four experimental groups using the =RAND() function in Microsoft Excel v.16 (Redmond, WA, USA): (1) intratumoral injections of VV-GMCSF-Lact (2.5 × 10^6^ PFU/mouse); (2) intraperitoneal injections of anti-PD-1 monoclonal antibodies (BioXCell, Lebanon, NH, USA; 100 μg/mouse); and (3) combination therapy, or intratumoral saline injections (control).

The virus was injected three times at 7-day intervals. Anti-PD-1 antibodies were administered in three treatment courses, each consisting of daily injections for three consecutive days, with 4-day intervals between courses. Mice in the combination therapy group received both treatments according to the same dosing schedule used for the respective monotherapy groups. Control animals received intratumoral saline injections.

Each mouse was considered an independent experimental unit. Animals were included if a visible tumor node was present at the transplantation site and the animal appeared healthy at the start of treatment. Exclusion criteria were the absence of a tumor node or signs of poor health. No animals met these exclusion criteria, and all eight mice per group were included in the final analysis. The order of treatment administration and tumor measurements was randomized daily.

Investigators involved in animal treatment were aware of group allocation. However, investigators performing RNA extraction and transcriptome analysis were blinded to group assignment.

### 4.4. RNA Sequencing

Total RNA was extracted from control GL261 tumors and tumors treated with anti-PD-1 antibodies, VV-GMCSF-Lact, or combination therapy using a standard TRIzol protocol. RNA integrity was assessed using an Agilent 2100 Bioanalyzer (Agilent Technologies, Santa Clara, CA, USA) with the RNA Pico Kit.

Polyadenylated RNA was enriched from 500 ng of total RNA using an NEBNext Poly(A) mRNA Magnetic Isolation Module (New England Biolabs, Ipswich, MA, USA). Directional cDNA libraries were prepared using the NEBNext Ultra II Directional RNA Library Prep Kit (New England Biolabs, Ipswich, MA, USA) according to the manufacturer’s instructions. Library fragment size distributions were analyzed using an Agilent 2100 Bioanalyzer with the High Sensitivity DNA Kit (Agilent Technologies), and library concentrations were quantified using a Qubit 2.0 Fluorometer (Thermo Fisher Scientific, Waltham, MA, USA) with the Qubit dsDNA HS Assay Kit.

High-throughput sequencing was performed on the FastaSeq300 platform (v2.0; GeneMind, Shenzhen, China), generating 75-nucleotide single-end reads. cDNA library preparation and sequencing were conducted at the Interdisciplinary Center for Shared Use of Kazan Federal University (Kazan, Russia).

### 4.5. Whole-Transcriptome Analysis

Raw FASTQ reads were quality-checked and aligned to a combined reference comprising the mouse genome (GRCm39/mm39) and the VV-GMCSF-Lact genome using STAR v2.7.11b [9] with the RefSeq gene annotation. Gene-level counts were generated using the quantMode GeneCounts option in STAR v2.7.1a [9].

Differential gene expression analysis was performed using DESeq2 v1.36.0 with standard parameters [10] within the Bioconductor v3.14 framework. For each pairwise comparison (control vs. VV-GMCSF-Lact, control vs. anti-PD-1, and control vs. combination), we compiled lists of differentially expressed transcripts using a Benjamini–Hochberg adjusted *p*-value < 0.05 and |log2FoldChange| > 0.5. The top 300 genes displayed in Venn diagrams represent the maximum number of genes meeting these inclusion criteria across all three comparisons. This cutoff was chosen to standardize visualization across treatment groups while capturing the most robust transcriptional changes.

Significantly upregulated and downregulated gene sets were analyzed using Enrichr [11] via the R package enrichR v3.4. The following libraries were queried for functional enrichment analysis: “GO Biological Process 2025”, “KEGG 2019 Mouse”, “KEGG 2021 Human”, “MSigDB Hallmark 2020”, and, for transcription factor-associated gene sets, “ENCODE and ChEA Consensus TFs from ChIP-X” and “TRRUST Transcription Factors 2019”. Pathways with adjusted *p*-values < 0.05 were considered significantly enriched.

To investigate potential therapy-associated changes in TME composition, relative cell-type contributions were estimated using DeconRNASeq v1.50.0 [12], with a reference panel of 54 mouse transcriptomes from the NCBI SRA database (Table S2). For cell types represented by multiple reference samples, the highest non-zero value was selected. This approach ensured consistent comparison of the same predefined transcriptional reference patterns across all treatment groups, yielding relative rather than absolute estimates of cell-type contributions. This strategy prioritized comparability across treatment conditions over precise compositional inference.

All statistical analyses and visualizations were performed in R v4.5.2 (R Core Team, 2023).

## 5. Conclusions

Whole-transcriptome analysis of subcutaneously transplanted GL261 glioma tumors in immunocompetent C57BL/6 mice demonstrates that both VV-GMCSF-Lact oncolytic virotherapy and anti-PD-1 immune checkpoint blockade substantially remodel the glioma tumor microenvironment. Transcriptomic profiling indicates that anti-PD-1-containing regimens are the dominant drivers of global transcriptional reprogramming, whereas VV-GMCSF-Lact monotherapy induces a more heterogeneous but immunologically active response. This response is characterized by increased expression of genes associated with T cell and B cell activation, NKT cell signaling, suppression of tumor-associated proliferative programs, and a decreased proportion of malignant cells.

Despite these differences, all treatment modalities converge on a shared proinflammatory transcriptional program involving IFNG signaling, TNF-alpha/NF-kB activation, and induction of adaptive immune resistance markers such as PD-L1. These results support a model in which both virotherapy and immune checkpoint blockade activate common inflammatory pathways within the TME while simultaneously triggering compensatory immunosuppressive feedback. The consistent suppression of EMT-associated genes across all treatment groups further suggests that immune-mediated therapeutic pressure may reduce invasive and aggressive tumor phenotypes independently of treatment modality.

Importantly, combination therapy with VV-GMCSF-Lact and anti-PD-1 antibodies induces unique transcriptional programs not detected with either monotherapy. Particularly, it promotes the upregulation of monocyte chemotaxis-related genes, including *Ccl2*, *Cxcl1*, *Cxcl5*, and *Mmp8*. These changes suggest enhanced coordination between the adaptive and innate immune compartments within the TME. In parallel, modulation of immune-regulatory phosphatase genes, including downregulation of *Ppp2r1a/b* and modulation of *Ptpn6* and *Ctla4*, indicates that combination therapy may reshape inhibitory signaling networks that control T cell activation and exhaustion. Selective suppression of the Rap1 signaling pathway, together with downregulated EMT-associated programs, may reflect attenuation of molecular processes linked to glioma cell migration and invasiveness.

Overall, our study provides insight into how VV-GMCSF-Lact and PD-1 blockade remodel the glioma immune microenvironment and demonstrates that their combination induces transcriptional programs not observed with either monotherapy. This supports the rationale for combining oncolytic vaccinia virotherapy with immune checkpoint inhibition for glioma treatment. Further validation will be required to confirm these molecular programs and determine their contribution to therapeutic efficacy.

## Figures and Tables

**Table 1:** RNA-seq data characteristics of GL261 glioma tumors subcutaneously transplanted into C57BL/6 mice after treatment with anti-mouse PD1 IgG antibodies (anti-PD-1), the oncolytic virus VV-GMCSF-Lact, or their combination.

Sample	Total Number of Sequencing Reads	Reads Mapped to the Mouse Genome mm39 (%)	Gender	Tumor Treatment
Cm1	2.00 × 10^7^	95.70	Male	Control (Placebo)
Cm2	2.10 × 10^7^	95.37		
Pf1	2.05 × 10^7^	96.41	Female	anti-PD-1 IgG (Antibodies to PD1)
Pf2	2.46 × 10^7^	96.76		
Vm1	2.11 × 10^7^	94.74	Male	VV-GMCSF-Lact (Oncolytic virus)
Vm2	1.90 × 10^7^	95.01		
VPf1	1.36 × 10^7^	96.87	Female	VV-GMCSF-Lact + anti-PD-1 (Combination of anti-PD-1 and oncolytic virus)
VPm2	1.66 × 10^7^	96.39	Male	

## Data Availability

RNA-seq data have been deposited in the NCBI SRA database under accession code PRJNA1514699. Other data supporting the findings of this study are available in the article and its Supplementary Files. Further inquiries can be directed to the corresponding authors.

## Supplementary Materials

The following supporting information can be downloaded at https://www.mdpi.com/article/10.3390/ijms27188078/s1.

## Abbreviations

The following abbreviations are used in this manuscript:

CAF	Cancer-associated fibroblast
EMT	Epithelial–mesenchymal transition
GBM	Glioblastoma multiforme
IFNG	Interferon gamma
MDSC	Myeloid-derived suppressor cell
NK	Natural killer cell
NKT	Natural killer T cell
PD-1	Programmed death receptor 1
PD-L1	Programmed death ligand 1
TAN	Tumor-associated neutrophil
TFs	Transcription factors
TME	Tumor microenvironment
Treg	Regulatory T cell

## References

[1] Mirimanoff R.-O., Gorlia T., Mason W., Van Den Bent M.J., Kortmann R.-D., Fisher B., Reni M., Brandes A.A., Curschmann J., Villa S. (2006). Radiotherapy and Temozolomide for Newly Diagnosed Glioblastoma: Recursive Partitioning Analysis of the EORTC 26981/22981-NCIC CE3 Phase III Randomized Trial. J. Clin. Oncol..

[2] Perez A., Huse J.T. (2021). The Evolving Classification of Diffuse Gliomas: World Health Organization Updates for 2021. Curr. Neurol. Neurosci. Rep..

[3] Lira G.A., Brandão J.D.A., Anderson L., Bassi Ê.J. (2025). Immune Checkpoint Inhibitors in Cancer Patients from the Perspective of Pharmaceutical Care: A Scoping Review. Int. J. Pharm. Pract..

[4] Yang T., Kong Z., Ma W. (2021). PD-1/PD-L1 Immune Checkpoint Inhibitors in Glioblastoma: Clinical Studies, Challenges and Potential. Hum. Vaccines Immunother..

[5] Tritz Z.P., Ayasoufi K., Johnson A.J. (2021). Anti-PD-1 Checkpoint Blockade Monotherapy in the Orthotopic GL261 Glioma Model: The Devil Is in the Detail. Neuro-Oncol. Adv..

[6] Ageenko A.B., Vasileva N.S., Chesnokova A.S., Semenov D.V., Byvakina A.A., Dymova M.A., Sen’kova A.V., Nushtaeva A.A., Leonteva A.A., Savinovskaya Y.I. (2026). Oncolytic Virus VV-GMCSF-Lact and Human GM-CSF Against GL261 Glioma in Immunocompetent Mice. Pharmaceuticals.

[7] Semenov D., Vasileva N., Menyailo M., Mishinov S., Savinovskaya Y., Ageenko A., Chesnokova A., Dymova M., Stepanov G., Kochneva G. (2025). Single-Cell Transcriptomic Changes in Patient-Derived Glioma and U87 Glioblastoma Cell Cultures Infected with the Oncolytic Virus VV-GMCSF-Lact. Int. J. Mol. Sci..

[8] Ageenko A., Vasileva N., Richter V., Kuligina E. (2024). Combination of Oncolytic Virotherapy with Different Antitumor Approaches against Glioblastoma. Int. J. Mol. Sci..

[9] Dobin A., Davis C.A., Schlesinger F., Drenkow J., Zaleski C., Jha S., Batut P., Chaisson M., Gingeras T.R. (2013). STAR: Ultrafast Universal RNA-Seq Aligner. Bioinformatics.

[10] Love M.I., Huber W., Anders S. (2014). Moderated Estimation of Fold Change and Dispersion for RNA-Seq Data with DESeq2. Genome Biol..

[11] Chen E.Y., Tan C.M., Kou Y., Duan Q., Wang Z., Meirelles G.V., Clark N.R., Ma’ayan A. (2013). Enrichr: Interactive and Collaborative HTML5 Gene List Enrichment Analysis Tool. BMC Bioinform..

[12] Gong T., Szustakowski J.D. (2013). DeconRNASeq: A Statistical Framework for Deconvolution of Heterogeneous Tissue Samples Based on mRNA-Seq Data. Bioinformatics.

[13] Semenov D.V., Vasileva N.S., Dymova M.A., Mishinov S.V., Savinovskaya Y.I., Ageenko A.B., Dome A.S., Zinchenko N.D., Stepanov G.A., Kochneva G.V. (2023). Transcriptome Changes in Glioma Cells upon Infection with the Oncolytic Virus VV-GMCSF-Lact. Cells.

[14] Oropeza-Martínez E., Palacios Serrato E.G., Zamora-Salas S.X., Lira-Rodríguez N.A., López-Mignon S.H., Martinez-Benitez M.B., Tecalco-Cruz A.C. (2025). Interferon-Gamma Signaling Pathway: Modulation of Key Genes in the Progression of Glioblastoma. World J. Biol. Chem..

[15] Garcia-Diaz A., Shin D.S., Moreno B.H., Saco J., Escuin-Ordinas H., Rodriguez G.A., Zaretsky J.M., Sun L., Hugo W., Wang X. (2017). Interferon Receptor Signaling Pathways Regulating PD-L1 and PD-L2 Expression. Cell Rep..

[16] Takacs G.P., Kreiger C.J., Luo D., Tian G., Garcia J.S., Deleyrolle L.P., Mitchell D.A., Harrison J.K. (2023). Glioma-Derived CCL2 and CCL7 Mediate Migration of Immune Suppressive CCR2+/CX3CR1+ M-MDSCs into the Tumor Microenvironment in a Redundant Manner. Front. Immunol..

[17] Zhong Y., Zhang W., Zheng C., Wu H., Luo J., Yuan Z., Zhang H., Wang C., Feng H., Wang M. (2025). Multi-Omic Analyses Reveal PTPN6’s Impact on Tumor Immunity across Various Cancers. Sci. Rep..

[18] Zhang Y.-L., Wang R.-C., Cheng K., Ring B.Z., Su L. (2017). Roles of Rap1 Signaling in Tumor Cell Migration and Invasion. Cancer Biol. Med..

[19] Zamora-Salas S.X., Macías-Silva M., Tecalco-Cruz A.C. (2024). Upregulation of the Canonical Signaling Pathway of Interferon-Gamma Is Associated with Glioblastoma Progression. Mol. Biol. Rep..

[20] Karri V., Dalia S.M. (2025). Persistent IFN-γ Signaling in Acquired Resistance to PD-(L)1 Blockade in NSCLC. J. Transl. Genet. Genom..

[21] Lin D., Shen Y., Liang T. (2023). Oncolytic Virotherapy: Basic Principles, Recent Advances and Future Directions. Signal Transduct. Target. Ther..

[22] Huang R.-Y., Francois A., McGray A.R., Miliotto A., Odunsi K. (2017). Compensatory Upregulation of PD-1, LAG-3, and CTLA-4 Limits the Efficacy of Single-Agent Checkpoint Blockade in Metastatic Ovarian Cancer. OncoImmunology.

[23] Xia P. (2026). The Significance of Epithelial–Mesenchymal Transition (EMT) in the Initiation, Plasticity, and Treatment of Glioblastoma. Genes Dis..

[24] Colardo M., Segatto M., Di Bartolomeo S. (2021). Targeting RTK-PI3K-mTOR Axis in Gliomas: An Update. Int. J. Mol. Sci..

[25] Kennel K.B., Bozlar M., De Valk A.F., Greten F.R. (2023). Cancer-Associated Fibroblasts in Inflammation and Antitumor Immunity. Clin. Cancer Res..

[26] Narla G., Sangodkar J., Ryder C.B. (2018). The Impact of Phosphatases on Proliferative and Survival Signaling in Cancer. Cell. Mol. Life Sci..

[27] Clark M.C., Lu R.O., Ho W.S., Dias M.H., Bernards R., Forman S.J. (2024). A Combination of Protein Phosphatase 2A Inhibition and Checkpoint Immunotherapy: A Perfect Storm. Mol. Oncol..

[28] Teft W.A., Chau T.A., Madrenas J. (2009). Structure-Function Analysis of the CTLA-4 Interaction with PP2A. BMC Immunol..

[29] Rozemond H. (1986). Laboratory Animal Protection: The European Convention and the Dutch Act. Vet. Q..

