# Supplementary material for: Whole-Transcriptome Profiling of Murine GL261 Glioma Reveals Signatures Consistent with Distinct Immune Reprogramming Induced by Oncolytic Virotherapy with VV-GMCSF-Lact and Anti-PD-1 Therapy
Source: Int J Mol Sci. 2026 Sep 11;27(18):8078. doi: 10.3390/ijms27188078 (PMC13606747; doi:10.3390/ijms27188078)
Supplement: Supplementary file 1 [file ijms-27-08078-s001.zip › ijms-4479963-supplementary.pdf]

**Table S1.** Changes in the cellular composition of subcutaneously transplanted glioma tumors following treatment with the oncolytic virus VV-GMCSF-Lact (VV), anti-PD-1 antibodies (A'PD1), or their combination (VV + A'PD1). Data were obtained using DeconRNASeq V1.50.0 and reference transcriptome arrays of mouse cells from the NCBI SRA archive (Table S2).

| Reference Cells | Description | SRA ID | Mean (%) | | | |
|---|---|---|---|---|---|---|
| | | | Ctrl | A'PD1 | VV | VV + A'PD1 |
| GL261 | Murine glioma | SRR3063569 | 42.2 | 38.4 | 31.7 | 40.0 |
| Treg | Regulatory T cells | GSM8245724 | 33.5 | 32.5 | 25.6 | 27.9 |
| CAFs | Cancer associated fibroblasts | GSM7106656 | 5.1 | 9.4 | 10.8 | 10.5 |
| CD8 T cells | Cytotoxic T lymphocytes | GSM8561399 | 6.8 | 9.1 | 11.4 | 8.2 |
| TDEC | Tumor-derived endotheliocy tes | GSM5974192 | 10.2 | 7.7 | 7.3 | 8.7 |
| NKT | Natural killer T cells | GSM8344661 | 1.5 | 0.4 | 6.6 | 2.6 |
| MDSC | Myeloid-derived suppressor cells | GSM7666312 | 0.2 | 2.5 | 4.0 | 1.9 |
| B cells | B lymphocytes | GSM8377210 | 0.5 | 0.0 | 1.3 | 0.2 |
| CD4 T cells | Helper T cell | GSM8430295 | 0.0 | 0.0 | 1.2 | 0.0 |

**Table S2.** Reference transcriptomes used for cell type deconvolution.

| SRA ID | Cell type |
|---|---|
| GSM8245724 | Treg |
| SRR3063569 | GL261 |
| SRR5457920 | GL261 |
| GSM5974192 | TDEC |
| GSM7106656 | CAF |
| GSM8561399 | CD8 T cells |
| SRR21743141 | GL261 |
| GSM8548420 | Treg |
| GSM8073311 | Skin |
| GSM4808297 | TDEC |
| GSM8377286 | HSC |
| GSM7666312 | MDSC |
| GSM8344661 | NKT |
| GSM8574737 | Myocytes |
| GSM8020816 | Treg |
| GSM6225718 | NKT |

| | |
|---|---|
| SRX26219314 | Macrophages |
| GSM8430804 | B cells |
| GSM8377252 | CD8 Memory T cell |
| GSM8698427 | Myocytes |
| SRR22308747 | Myocytes |
| GSM8377205 | Adipocytes |
| GSM7517708 | NKT |
| GSM8698418 | Myocytes |
| ERX10010770 | TANs |
| GSM6672279 | TANs |
| GSM6754057 | TANs |
| GSM7321008 | TANs |
| GSM7474811 | MDSC |
| GSM7885393 | CAF |
| GSM8073312 | Skin |
| GSM8073313 | Skin |
| GSM8375837 | NK |
| GSM8375838 | NK |
| GSM8377210 | B cells |
| GSM8377229 | Endothelial |
| GSM8377239 | CD4 Memory |
| GSM8377246 | CD4 Naive |
| GSM8377259 | CD8 Naive |
| GSM8377269 | CD8 T cells |
| GSM8377294 | Keratinocytes |
| GSM8377318 | Endothelial |
| GSM8377326 | Fibroblasts |
| GSM8377332 | Fibroblasts |
| GSM8377343 | Satellite stem cells |
| GSM8377349 | Fibroblasts |
| GSM8377358 | Epithelial |
| GSM8377367 | Fibroblasts |
| GSM8430295 | CD4 T cells |
| SRX26219313 | Macrophages |
| SRX26219313 | Macrophages |
| SRX26219314 | Macrophages |
| SRX26219315 | Macrophages |
| SRX26219315 | Macrophages |
